# Development of selective nanomolar cyclic peptide ligands as GBA1 enzyme stabilisers†

**DOI:** 10.1039/d4cb00218k

**Published:** 2025-01-31

**Authors:** Rebecca E. Katzy, Renier H. P. van Neer, Maria J. Ferraz, Kim Nicolai, Toby Passioura, Hiroaki Suga, Seino A. K. Jongkees, Marta Artola

**Affiliations:** a Department of Medical Biochemistry, Leiden Institute of Chemistry, Leiden University P.O. Box 9502 2300 RA Leiden The Netherlands m.e.artola@lic.leidenuniv.nl; b Department of Chemistry, Graduate School of Science, The University of Tokyo Tokyo Japan; c Chemical Biology and Drug Discovery, Utrecht Institute for Pharmaceutical Sciences, Utrecht University Utrecht 3584 CG The Netherlands S.A.K.Jongkees@vu.nl

## Abstract

The stabilisation of recombinant glycosidases by exogenous ligands, known as pharmacological chaperones or enzyme stabilisers, has recently garnered great clinical interest. This strategy can prevent enzyme degradation in the blood, reducing required dosages of recombinant enzyme and extending IV injection intervals, thereby reducing side effects, improving patient lifestyles and treatment costs. While this therapeutic approach has been successfully implemented for treating Pompe and Fabry diseases, clinical studies for Gaucher disease using chaperones alone or in combination with enzyme replacement therapy (ERT) have been limited, and no small molecule chaperones have yet been approved for this condition. Developing such therapies requires selective and effective reversible GBA1 ligands. Here, we describe the development of a new class of selective macrocyclic peptide GBA1 ligands using random nonstandard peptides integrated discovery (RaPID) technology, and demonstrate their ability to bind and stabilise rhGBA1 in plasma at nanomolar concentrations. These cyclic peptides do not inhibit endogenous GBA1 in cells due to poor cell permeability but can stabilise extracellular rhGBA1 in plasma, presenting significant potential as a combinatorial ERT-pharmacological chaperone therapy for Gaucher disease.

## Abstract 2

Macrocyclic peptide ligands developed *via* RaPID technology selectively bind and stabilize rhGBA1 in plasma at nanomolar concentrations without inhibiting endogenous GBA1, offering potential for combinatorial ERT-pharmacological chaperone for Gaucher disease.

## Graphical abstract

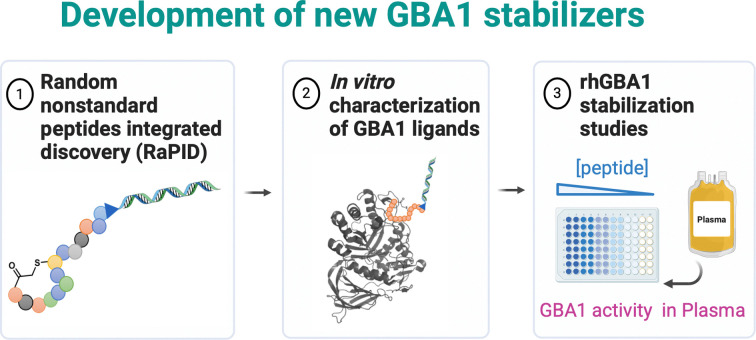

## Introduction

Lysosomal storage disorders (LSDs) are inherited diseases resulting from genetic defects that disrupt lysosomal function, including mutations in genes encoding lysosomal hydrolases, membrane proteins, and transporters. These defects cause impaired substrate metabolism, leating to the accumulation of metabolites such as glycosphingolipids.^1,2^ In particular, Gaucher disease (GD) is a genetic disease caused by deficiency in glucocerebrosidase (acid glucosylceramidase, GBA, EC 3.2.1.45) and is considered the most common autosomal recessive LSD, with approximately 1 : 100 000 incidence in the general population.^3–5^ GD has recently attracted global attention since mutations in the GBA1 gene have been demonstrated to be a genetic risk factor (about 20-fold) for developing Parkinson's disease (PD) and Lewy-body dementia, and thus patients with altered glucosylceramide (GlcCer) metabolism linked to heterozygotous GBA1 mutations do not suffer from GD but may actually develop these neuropathological disorders.^6–11^ In addition, GD patients have an increased risk of multiple myeloma, which is linked to chronic immune dysregulation caused by the antigenic properties of partially metabolized lysolipid substrates.^12,13^ GBA1 is responsible for the hydrolysis of GlcCer yielding glucose and ceramide. In cells of Gaucher patients, impaired hydrolysis of GlcCer leads to ongoing toxic accumulation of diverse metabolites inside lysosomes. Notably GlcCer and its sphingobase glucosylsphingosine (GlcSph), also known as lyso-Gb1, produced by acid ceramidase, are key hallmarks for disease progression.^14–16^ Lipid-laden macrophages in spleen, liver and bone-marrow are characteristic of Gaucher patients, and these cells secrete GlcSph, chitotriosidase and chemokine CCL18 to the plasma.^15,17,18^ It is well established that excessive GlcSph contributes to the symptomatology of Gaucher disease including chronic inflammation, bone loss, neurodegeneration.^8,19–22^ Different types of GD are described based on the severity of the clinical manifestations, ranging from the non-neuropathic GD type 1 with enlargement of spleen and liver as main symptoms to the more severe GD type 2 and 3 with neurological manifestations.^23^

GD patients can be treated with recombinant human GBA1 (rhGBA, Cerezyme®), known as enzyme replacement therapy (ERT), which ameliorates symptomatology but does not prevent neurological disease progression and/or autoimmunological responses.^24,25^ Cerezyme® is administered as an intravenous (IV) infusion over 1–2 hours every two weeks to Gaucher patients, requiring large amounts due to its limited half-life. In contrast to the administration of very expensive and not brain permeable recombinant enzyme, the use of relatively small molecules able to reduce the synthesis of GlcCer by inhibiting glucosylceramide synthase (GCS) is a therapeutic alternative.^26^ Miglustat and Eliglustat are two approved substrate reduction therapies (STR) for the treatment of GD type 1, although both suffer from limitations and side effects.^27^ Whereas Eliglustat treatment has demonstrated long-term safety and efficacy in GD type 1 patients,^28^ comparable to ERT,^29^ it neither crosses the blood–brain barrier nor ameliorates neurological symptoms.^30,31^ Venglustat, on the other hand, is a brain-penetrant GCS inhibitor that has shown acceptable safety, tolerability, and preliminary clinical efficacy in adults with type 3 Gaucher disease receiving Cerezyme.^32^ Gene therapy, using adeno-associated virus vectors able to deliver GBA1 gene and restore GBA1 activity, is currently under investigation, but with limited results.^33^ Another therapeutic strategy is to promote the correct folding of the endogenous mutated GBA1 and/or stabilise the recombinant enzyme used in ERT with small reversible molecules, known as pharmacological chaperone therapy, to prevent misfolding and assist the trafficking of the protein towards the lysosome.^34,35^ Several iminosugars including *N*-nonyl deoxynojirimycin (NN-DNJ),^36^ α-1-*C*-nonyl-1,5-dideoxy-1,5-iminoxylitol (α-1-*C*-Nonyl-DIX),^35^ isofagomine (IFG),^34,37^ bicyclic sp^2^-iminosugar nojirimycin (NJ),^38^l-idonojirimycin NAdBT-AIJ,^39,40^ and α-1-*C*-tridecyl-DAB have been shown to increase activity of GBA1 in Gaucher fibroblasts. However, some iminosugars present limited selectivity over other glycosidases and GCS. Ambroxol (ABX), a secretolytic agent used for the treatment of excessive mucus in newborns, is a structurally different weak GBA1 inhibitor that has shown to increase GBA1 activity by enhancing Saposin C and LIMP-2 protein levels, and is currently undergoing phase II clinical trials.^41–44^ Non-inhibitory chaperones such as NCGC758^45^ and S-181^46^ increase GBA1 activity and reduce lipid substrates and α-Syn levels in brain.^46,47^ More recently, the potent quinazoline allosteric modulator, JZ-4109, has been shown to induce GBA1 dimerization and to stabilise wild-type and N370S mutant GBA1 in patient-derived fibroblast cells.^48^ New structurally diverse GBA1 reversible inhibitors as potential stabilisers are of great interest for GD and PD. GBA1 stabilisers that can maintain GBA1 folding in circulation without penetrating the cell membrane or inhibiting the enzyme in cells would be a valuable addition to the current small molecule repertoire. Peptides are a class of molecules yet to be explored as ligands for GBA1. Random nonstandard peptides integrated discovery (RaPID) has emerged as a promising peptide-mRNA display technology able to screen libraries of >10^12^ macrocyclic peptides against diverse proteins.^49,50^ This methodology facilitates the discovery of potent constrained peptides, with some high affinity ligands currently under clinical investigations for the treatment of diverse diseases.^51^ Here, we illustrate how the RaPID methodology led to the identification of the first macrocyclic peptides as GBA1 ligands and describe their GBA1 binding and stabilisation properties, as well as their selectivity against other glycosidases.

## Results and discussion

### Library design, selection and synthesis of identified peptides

Since the primary goal of this work was to identify ligands for GBA, we sought a selection approach that would promote the enrichment of ligands that bind the protein in its natural environment. The interaction of GBA1 with the lysosomal membrane was mimicked through the addition of 0.2% sodium taurocholate,^52^ whereas the binding of GBA1 with activator protein Saposin C was replicated by addition of the interacting fragment Saposin C residues 41–60,^53,54^ prepared by solid phase peptide synthesis. The lysosomal conditions were mimicked by the use of McIlvaine buffer at pH 5.2, where GBA1 is most active.^55,56^ The protein was chemically biotinylated on lysines using NHS chemistry to allow for immobilisation on magnetic beads (Fig. S1, ESI†) and thereby efficient washing to remove non-binding library members. By carrying out this modification randomly across the protein surface we retain the ability to enrich binders across the protein surface in an unbiased way. We considered both active site binders and allosteric binders to be potentially valuable as stabilisers.

For the selection, we used two libraries of peptides macrocyclised from an N-terminal chloroacetyl-d- or -l-tyrosine to a C-terminal cysteine as a head-to-sidechain thioether (two separate selections, refered to as l-Tyr and d-Tyr selections, all other amino acids as canonical l-amino acids with methionine omitted to facilitate peptide initiation by *N*-chloroacetylated tyrosine by genetic code reassignment). These tyrosine and cysteine residues flank a random region of between 4 and 15 amino acids, encoded by NNK codons to improve amino acid balance and decrease the proportion of stop codons and mixed proportional to theoretical sequence diversity. Binders for the GBA1 target were enriched from this library over 5 sequential rounds of pull-down by immobilised target, amplification of the attached mRNA/cDNA tag, and library resynthesis by *in vitro* transcription and translation, at which point a large increase in recovery was oberved with rhGBA1 target but not with control magnetic beads (Fig. S2, ESI†).

**Fig. 1:**
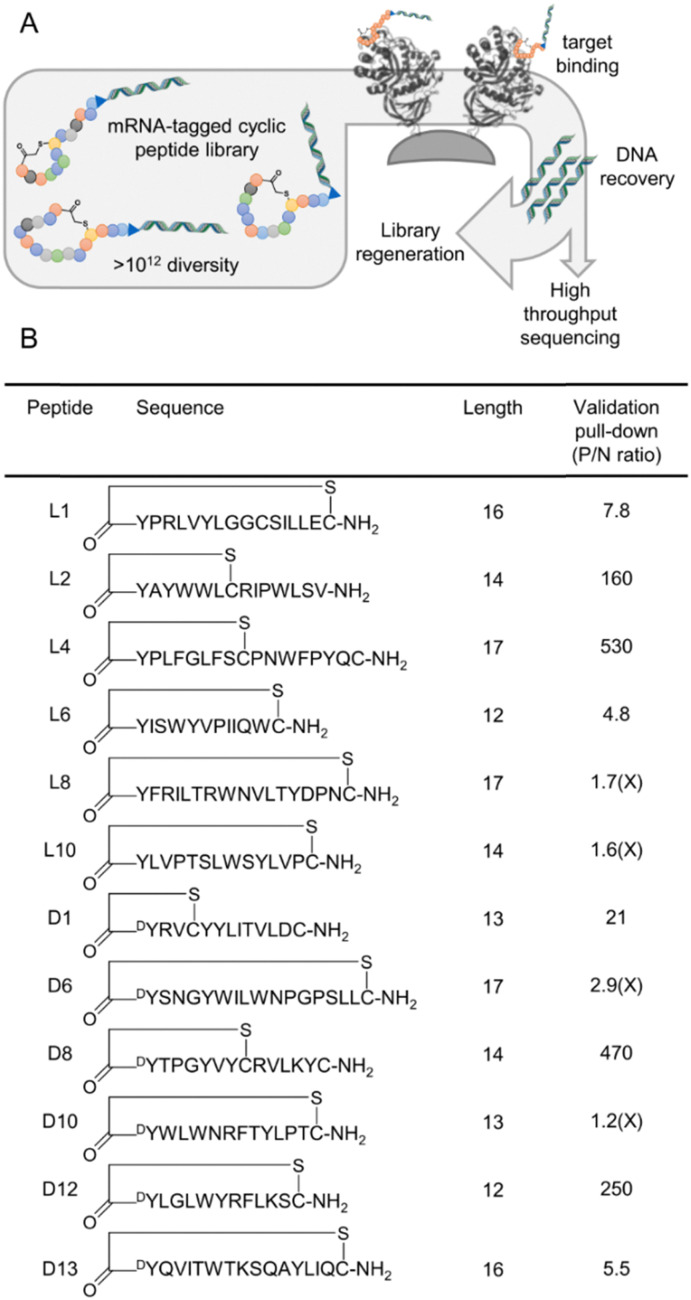
Selection of macrocyclic peptides binding to GBA. (A) Simplified schematic representation of the RaPID system, showing (i) peptide library generation through *in vitro* transcription, ligation, translation, and reverse transcription; enrichment of target-binding peptides through pull-down with bait protein immobilised on magnetic beads; (ii) recovery of DNA by PCR of cDNA; and (iii) either regeneration of library for the next selection round or sequencing of output DNA. (B) List of peptides chosen from high throughput sequencing results for validation by pull-down of individual sequence, showing peptide name, peptide sequence and semi-structural representation of cyclisation, peptide length, and peptide recovery in a validation pull-down of a single sequence (P/N ratio, positive to negative ratio; recovery using GBA1 on streptavidin magnetic beads *versus* recovery with streptavidin beads alone). All sequences except those indicated with ‘X’ in the final column were selected and synthesised on solid phase for further testing.

Deep sequencing was carried out for both enriched libraries to identify potential binders based on fractional enrichment in the final round. Six peptide sequences were chosen from each library for further investigation, based on maximised diversity (Fig. 1 and Fig. S2, ESI†). These peptides were validated by pull-down in the same selection system, from which two from each set were excluded because of poor binding. The remaining eight peptides (four from each library) were synthesized through routine Fmoc solid phase peptide synthesis. These peptides are referred to by the initiating amino acid stereochemistry and a number reflecting the abundance rank in the final enriched library. Within these hits, we found a diverse set of ring sizes and several lariat peptides with an additional cysteine arising in the random region (3/6 for the l-tyrosine library and 2/6 from the d-tyrosine library). One of these lacks the C-terminal cysteine hard-coded at the end of every sequence in the library, which likely was lost through a frameshift mutation as the C-terminal ‘GSGSGS’ spacer region has also mutated, but it appears to otherwise be a valid hit. The relatively high abundance of small rings may suggest that a small binding pocket in GBA1 is being exploited, or that the smaller macrocycle stabilises the conformation of the linear tail.^57^ Almost all hits contained at least one tryptophan and an additional (‘non-initiating’) tyrosine, suggesting aromatic interactions may be also important. However, the context for these aromatic residues was highly varied across the sequences and so we cannot draw any strong conclusions about structure–activity relationships.

### *In vitro* and *in situ* cell GBA1 Inhibition and selectivity

Based on the pull-down validation, a set of eight cyclic peptides (L1, L2, L4, L6, D1, D8, D12 and D13) were identified, synthesized, and evaluated for their inhibitory potency against recombinant human GBA1 (rhGBA1, Cerezyme®) and their selectivity over human β-glucosidases GBA2 and GBA3. Half-maximum inhibitory concentration (IC_50_) values for β-glucosidases were determined *in vitro* by measuring hydrolysis of 4-methylumbelliferyl-β-d-glucopyranoside (4-MU-β-d-Glu) fluorogenic substrate. All selected peptides inhibit rhGBA1 and L4, D1, and D12 proved to be low nanomolar inhibitors of GBA1, whereas D13 and L1 exhibited IC_50_ values in the high micromolar range (Table 1 and Fig. S3, ESI†). Kinetic studies showed that activity is constant with extended incubation periods, indicating that these peptides are reversible inhibitors and the obtained inhibitory constant (*K*_i_) values are in accordance with the IC_50_ results (Table 1 and Fig. S4, ESI†). The presence of competitive mixed inhibition behaviour suggests that the inhibitors likely have some affinity for the active site. Selectivity studies showed that all the peptides are completely selective for GBA1 and do not inhibit non-lysosomal GBA2 or GBA3, even at concentrations up to 100 μM.

**Table 1:** IC_50_ and *K*_i_ values for *in vitro* inhibition of rhGBA1 (Cerezyme), and IC_50_ values for GBA2 and GBA3 (using GBA2 or GBA3 overexpressing HEK293T lysates), respectively. Reported values are mean ± standard deviation from 3 technical triplicates. N.D.: not determined

Pept.	rhGBA1	rhGBA1	GBA2	GBA3
	IC_50_ (μM)	*K*_i_ (μM)	IC_50_ (μM)	IC_50_ (μM)
L1	138 ± 55.8	N.D.	>100	>100
L2	0.34 ± 0.16	0.042 ± 0.007	>100	>100
L4	0.009 ± 0.004	0.002 ± 0.001	>100	>100
L6	5.66 ± 2.04	1.34 ± 0.203	>100	>100
D1	0.005 ± 0.003	0.0009 ± 0.0001	>100	>100
D8	0.012 ± 0.011	0.013 ± 0.004	>100	>100
D12	0.004 ± 0.004	0.001 ± 0.0004	>100	∼85
D13	26.4 ± 3.31	N. D.	>100	N. D.

**Fig. 2:**
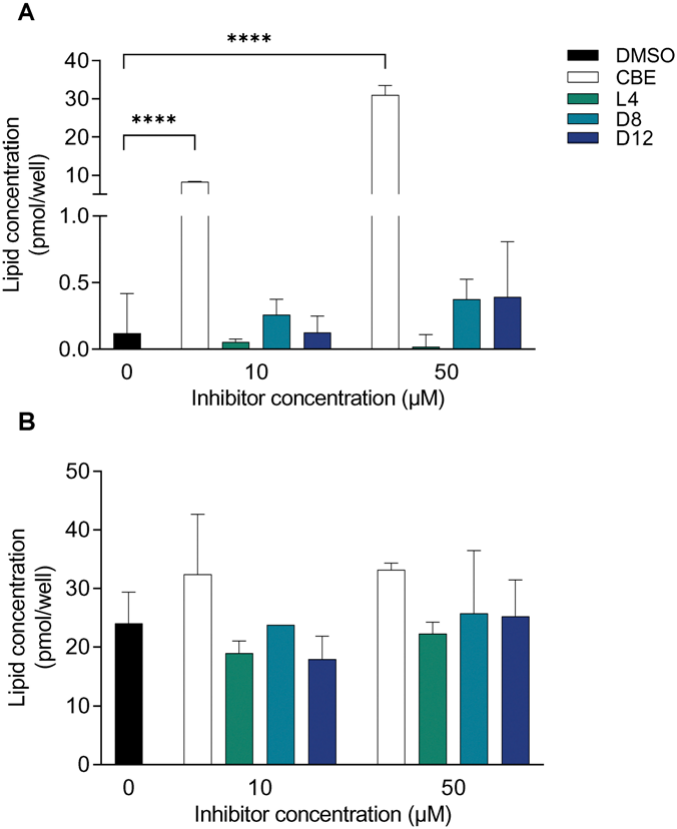
Glucosylsphingosine (A) and glucosylceramide (B) levels produced in fibroblasts treated with CBE, L4, D8 or D12 at 10 or 50 μM for 16 h. Error ranges depict standard deviations from *n* = 3 (biological replicates). **** *p* < 0.0001.

We further investigated GBA1 inhibition in cells. Human dermal fibroblasts were initially incubated with peptide concentrations ranging from 0.1 μM (from 0.001 μM for L4, D1 and D12) to 100 μM of each cyclic peptide for 3 hours. Notably, with the exception of D8 and D12, peptide concentrations above 10 μM showed toxicity to the cells. Most of the cells incubated with 100 μM peptide, not including D8 and D12, were partially detached and had a large amount of clumping. In addition, large granules were observed in fibroblasts incubated with peptides at 10 μM and 100 μM, including the ones incubated with D8 and D12, though these cells were still attached and otherwise looked healthy. Importantly, concentrations lower than 10 μM did not exhibit any toxicity, and the cells maintained morphology similar to the control. Given that *in situ* inhibition of GBA1 in cells leads to an increase in GlcSph levels due to the compensatory hydrolysis of accumulated GlcCer by acid ceramidase in the lysosome, we investigated GlcCer and GlcSph levels in fibroblasts to assess the *in situ* activity of L4, D8, and D12 (at 10 or 50 μM) after overnight incubation (Fig. 2). Unlike conduritol B epoxide (CBE), a well-established GBA1 inhibitor, none of the peptides significantly increased GlcSph levels. This suggests that, after overnight incubation at these concentrations, the peptides are unable to cross the cell membrane, reach the lysosomes of fibroblasts, inhibit GBA1, and consequently increase GlcCer and GlcSph levels. However, fibroblasts treated with the peptides overnight did exhibit GBA1 inhibition after cell lysis (Table S1, ESI†). To further confirm the inability of these peptides to cross the cell membrane, we conducted *in situ* competitive activity-based protein profiling (cABPP) experiments using RAW 264.7 monocyte/macrophage-like cells. These cells, known for their phagocytic and endocytic capabilities, typically demonstrate more efficient uptake of inhibitors. RAW 264.7 cells were then treated with increasing concentrations of cyclic peptides (0, 0.1, 1, 10, and 100 μM) for 4 hours, followed by exposure to a cell-penetrant Bodipy red broad spectrum GBA activity-based probe (ABP), JJB75^58^ at 10 nM for 2 hours (Fig. S5 for chemical structures, ESI†). After washing, the cell lysates were analysed by fluorescence SDS-gel scanning, which showed no significant competition, whereas the GBA1 ABP functionalized with a green Bodipy (MDW933^56^ as positive control) did show competition. This cABPP experiment further confirms that the peptides are unable to cross the cell membrane (Fig. S6, ESI†). Consistent with these findings, and unlike CBE, GlcSph and GlcCer levels produced by RAW 264.7 cells after 16 hours incubation with L1, L2, L4, L6, D1, D8, and D12 did not indicate GBA1 inhibition in cells (Fig. S7, ESI†).

### Enzyme stabilisation studies

We next studied the stabilisation of rhGBA1 (using Cerezyme® at a 1 μM concentration for an optimal signal) by the most potent inhibitors (L2, L4, D1, D8, and D12) using a label-free differential scanning fluorimeter (Table 2). The stabilisation effect was determined by measuring protein's structural integrity or unfolding profile in a label-free manner, assessing the fluorescence of intrinsic tryptophan and tyrosine residues detected aboth 350 nm (tryptophan) and 330 nm (tyrosine) while increasing the temperature (from 35 to 95 °C in 3 min run) in the presence or absence of increasing concentrations of peptides (Fig. S9, ESI†). These thermostability assays showed that L4, D8 and D12 stabilise rhGBA1 more efficiently than the well-documented GBA1 chaperone Ambroxol, with maximum shifts in inflection temperatures (Δ*T*_i,max_) of 13.4, 17.9 and 12.5 °C and half-maximal effective concentrations of 2.5, 2.2 and 1.9 μM, respectively (Table 2).

**Table 2:** Thermostability constants (Δ*T*_i,max_ and EC_50_) in recombinant human GBA1 (rhGBA1 at 1 μM). N. D.: not determined due to a lack of stabilisation effect

Compound	Δ*T*_i,max_ (°C)	EC_50_ (μM)
Ambroxol	12.3 ± 5.4	7.7 ± 4.9
L2	4.8 ± 0.3	2.0 ± 0.5
L4	13.4 ± 2.0	2.6 ± 1.1
D1	N. D.	N. D.
D8	17.9 ± 1.1	2.2 ± 0.5
D12	12.5 ± 1.1	1.9 ± 0.4

To further investigate the stabilisation potential of the peptides, we first evaluated the stability of Cerezyme® in plasma of healthy individuals and found that, while it is stable in McIlvaine buffer, it degrades in plasma with a half-life of 25 minutes for a 10 nM enzyme dilution (Fig. S10, ESI†). Notably, a more concentrated enzyme solution (25 nM) exhibited higher stability, likely aided by the additives present in the Cerezyme formulation. We then investigated whether peptides L4, D8, and D12 could stabilise rhGBA1 in plasma. For this, rhGBA1 at 10 nM was incubated in plasma with increasing concentrations of peptides (0, 0.001, 0.01, 0.1, 1, and 10 μM) or Ambroxol for comparison (100, 250, 500, and 1000 μM) for different time intervals (0, 15, 30, 45, 60, 75, 90, 105, and 120 minutes), all in the presence of 0.5% of DMSO. The solutions were then incubated with 4-MU-β-d-Glu for an additional 30 minutes, and the remaining rhGBA1 activity was determined. The control samples containing 0.5% DMSO showed that, on average, 81.1% of rhGBA1 degraded after 1 hour in plasma.

This degradation could be prevented or delayed in the presence of peptides D8 and D12, whereas L4 and Ambroxol showed no stabilisation effect under these conditions. Specifically, high concentrations of all peptides (10–1 μM) led to enzyme binding but also GBA1 inhibition after short incubations (0 to 30 minutes). Interestingly, 0.1 μM solutions of peptides D12 and D8 stabilised rhGBA1 over time. Specifically, 48.6% of enzyme activity remained after 60 minutes of incubation with D8, compared to 18.9% in the control sample. Additionally, 38.0% of enzyme activity was observed after 90 minutes of incubation with D8, compared to 5.3% in the control sample, demonstrating that co-administration of ERT with these peptide-based ligands slows down enzyme degradation in plasma. To discard potential degradation of D8 and D12 during plasma experiments, the peptides were incubated in plasma at 0.1 μM for 2 hours, followed by the addition of rhGBA1 to measure its activity. This was compared to controls where D8 and D12 were added to plasma and directly tested for GBA1 activity. The results demonstrated that both the peptides retained their inhibitory activity against rhGBA1 after 2 h pre-incubation in plasma, suggesting that they are not significantly degraded under these conditions (Fig. S11, ESI†).

**Fig. 3:**
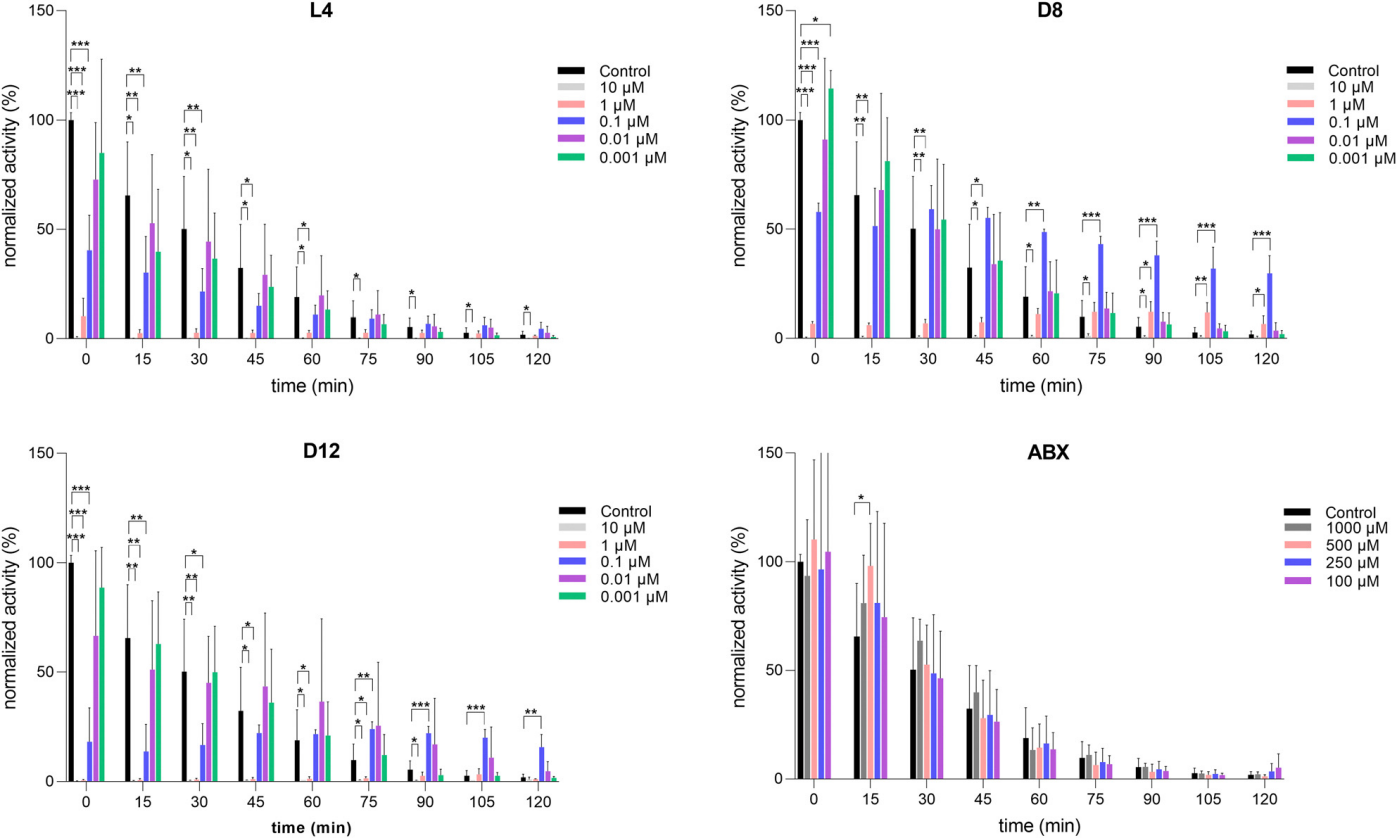
Stabilisation of rhGBA1 (10 nM) in plasma by cyclic peptides D8 and D12. Activity of rhGBA1 (10 nM) in plasma, represented as normalized activity (%) to the *t* = 0 condition with no inhibitor present, was measured in the presence of L4, D8, D12 and Ambroxol (ABX) at different concentrations and time incubations. Control contained 0.5% DMSO. Error ranges depict standard deviations from *n* = 3 replicates, measured in technical duplicates. *p < 0.05, **p < 0.01, ***p < 0.001.

**Fig. 4:**
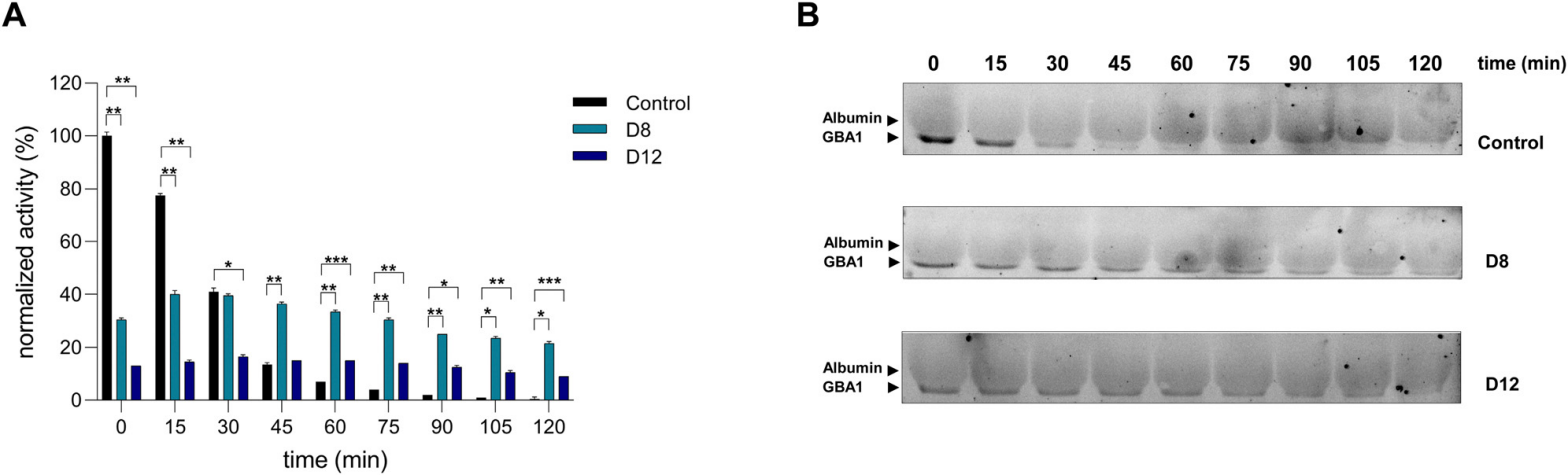
Stabilisation of rhGBA1 (10 nM) in plasma by cyclic peptides D8 and D12 at 0.1 μM: enzyme activity measured with 4-MU β-glucosise substrate compared to activity-based protein profilling. rhGBA1 (10 nM) was incubated in plasma in the presence D8 or D12 at different concentrations and time incubations (with 0.5% DMSO as control) and activity was anaylised using (A) 4-MU activity assays representing normalized activity (%) to the *t* = 0 condition with no inhibitor present. Error ranges depict standard deviations from *n* = 2 replicates, measured in technical duplicates. *p < 0.05, **p < 0.01, ***p < 0.001; (B) activity-based protein profiling (ABPP) using SDS-PAGE gel electrophoresis to visualise fluorescently labelled rhGBA1 with a broad spectrum Bodipy red GBA1, GBA2 and GBA3 (ABP JJB75 at 10 nM).

We further validated the rhGBA1 stabilization effect of the most promising peptides using activity-based protein profiling (ABPP) analysis. Recombinant human GBA1 (rhGBA1) was incubated in plasma with or without the peptides D8 and D12 at 0.1 μM, previously identified as optimal concentrations (Fig. 3). Samples were analysed using 4MU activity assays and ABPP coupled with SDS-PAGE gel electrophoresis to visualize labelled rhGBA1 by fluorescent scanning. Consistent with previous results, the 4MU assay demonstrated an initial decrease in rhGBA1 activity due to peptide binding and subsequent enzyme inhibition. However, rhGBA1 activity was preserved over time, reflecting the stabilizing effects of the peptides (Fig. 4A). Similarly, ABPP labelling with the broad-spectrum β-glucosidase probe JJB75, functionalized with a red Bodipy fluorophore, corroborated these findings. While quantification of ABP-labelled bands was challenging due to overlapping signals from abundant plasma albumin at a slightly higher molecular weight, the gel clearly showed that the rhGBA1 band persisted for up to 120 minutes when incubated with D8 at 0.1 μM. In contrast, the band disappeared after 45 minutes in the absence of peptides. These observations provide compelling evidence that D8 and in some extend also D12, significantly stabilize rhGBA1 in plasma over time.

## Conclusions

The stabilisation of recombinant glycosidases by exogenous ligands, known as pharmacological chaperones, has recently achieved clinical success. The first approved combination therapy, Opfolda® (Miglustat) and Pombiliti® (cipaglucosidase alfa) from Amicus Therapeutics, entered the European market in 2023 for the treatment of Pompe disease. This strategy can prevent enzyme degradation in the blood, thereby reducing the required dosages of recombinant enzyme or extending IV injection intervals. This not only improves patients' quality of life but also reduces side effects and treatment costs. To date, clinical studies for treating Gaucher disease with chaperones alone or in combination with ERT have been limited.^59,60^ No small molecule chaperones have yet been approved for the treatment of Gaucher disease. Developing such therapies requires selective and effective reversible GBA1 ligands. Although Ambroxol has been shown to reach the central nervous system and exert a stabilisation effect on GBA1, no randomized studies in large and diverse populations of patients with Gaucher disease have been undertaken.

Here, we describe the development of a new class of GBA1 ligands using mRNA display under a reprogrammed genetic code (RaPID technology) and demonstrate their ability to bind and stabilise rhGBA1. In particular, these peptides inhibit rhGBA1 *in vitro* at nanomolar concentrations, but we did not observe elevated GlcCer or GlcSph levels upon treatment of RAW 264.7 cells or human fibroblasts, suggesting they have poor cell permeability and thus do not inhibit GBA1 in cells. Competitive ABPP experiments further confirmed the lack of *in situ* cell GBA1 activity. With potent and non-cell-permeable GBA1 ligands in hand, we decided to explore their ability to stabilise rhGBA1 at high temperatures. Specifically, D8 and D12 improve GBA1 thermostability and prevent rhGBA1 degradation and increase rhGBA1 activity in plasma. Although the observed enzyme inhibition at high ligand concentrations and the weak competition using GBA1 activity-based probes in cells suggest orthosteric binding, whether the enzyme stabilisation is due to active site occupancy or allosteric activation remains to be investigated. Attempts to crystallize rhGBA1 in complex with D8 and D12 were unsuccessful, and further structural studies using for example CryoEM could shed light on their binding motifs. Further studies are needed to evaluate the effect of ERT in combination with cyclic peptides D8 and D12 in fibroblasts from Gaucher patients with diverse mutations and in animal models.

## Author contributions

M. A. and S. A. K. J. conceived and designed the investigations, interpreted experimental data, wrote and proofread the manuscript. R. E. K., M. J. F. and K. N. conducted enzymatic activity assays, lipidomics, enzyme stabilisation studies and interpreted experimental data under the supervision of M. A. R. H. P. v. N. performed protein binding tests, made the saposin blocking peptide, did the selection, analysed the sequencing, and did the first round of solid phase synthesis supervised by T. P. and H. S. and S. A. K. J.

## Data availability

The authors declare that all data supporting the findings of this study are available within the article and ESI,† and raw data files are available from the corresponding author upon request.

## Conflicts of interest

There are no conflicts to declare.

## Supplementary Material

CB-OLF-D4CB00218K-s001
