# Supplementary material for: Development of selective nanomolar cyclic peptide ligands as GBA1 enzyme stabilisers
Source: RSC Chem Biol. 2025 Jan 31;6(4):563–70. doi: 10.1039/d4cb00218k (PMC11808397; doi:10.1039/d4cb00218k)
Supplement: CB-006-D4CB00218K-s001 [file CB-006-D4CB00218K-s001.pdf]

Supplementary Information (SI) for RSC Chemical Biology.
This journal is © The Royal Society of Chemistry 2025

Supporting Information for

Development of Selective Nanomolar Cyclic Peptide Ligands as GBA1 Enzyme Stabilizers

Rebecca E. Katzy,[a] Renier H. P. van Neer,[b] Maria J. Ferraz,[a] Kim Nicolai,[a] Toby Passioura,[b] Hiroaki Suga,[b] Seino A. K. Jongkees,*[cd] and Marta Artola*[a]

[a] Department of Medical Biochemistry, Leiden Institute of Chemistry, Leiden University, P.O. Box 9502, 2300 RA Leiden, The Netherlands.

[b] Department of Chemistry, Graduate School of Science, The University of Tokyo, Tokyo, Japan

[c] Chemical Biology and Drug Discovery, Utrecht Institute for Pharmaceutical Sciences, Utrecht University, Utrecht 3584 CG, the Netherlands. E-mail: S.A.K.Jongkees@vu.nl

[d] *Current address:* Department of Chemistry and Pharmaceutical Sciences, Vrije Universiteit Amsterdam, De Boelelaan 1108, 1081 HZ Amsterdam, The Netherlands

Corresponding authors: S.A.K.Jongkees@vu.nl; m.e.artola@lic.leidenuniv.nl

TABLE OF CONTENTS

1. **Supporting Figures and Tables** S3
2. **Materials and Methods** S12
   - 2.1. Immobilisation of GBA on streptavidin beads S12
   - 2.2. Macrocyclic Peptide Library Design S12
   - 2.3. RaPID selection S12
   - 2.4. Peptide synthesis S13
   - 2.5. *In vitro* IC_{50} and K_i measurements S14
   - 2.6. *In situ* cell studies S15
   - 2.7. GlcCer and GlcSph lipid analysis S15
   - 2.8. Thermostability assays in rhGBA (Cerezyme) S15
   - 2.9. Cerezyme Stabilization studies in plasma S16
3. **Peptides Characterization** S17
4. **Raw gel images** S21
5. **References** S22

1. Supporting Figures and Tables

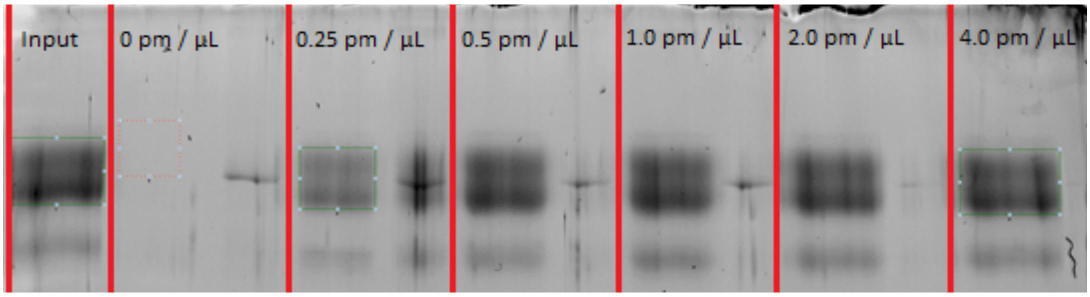

Figure S1. SDS-PAGE gel of biotinylated GBA1 immobilisation. 2 pmol of chemically biotinylated GBA was incubated with varied quantities of magnetic beads to find the saturation point (between 0.25 and 4 pmol.µL^{-1} bead slurry, with 0.25 pmol.µL^{-1} used throughout the selection to ensure an excess of protein relative to streptavidin beads and thereby minimise free binding sites). Each pair of lanes represents the unbound and bead-bound fractions, respectively.

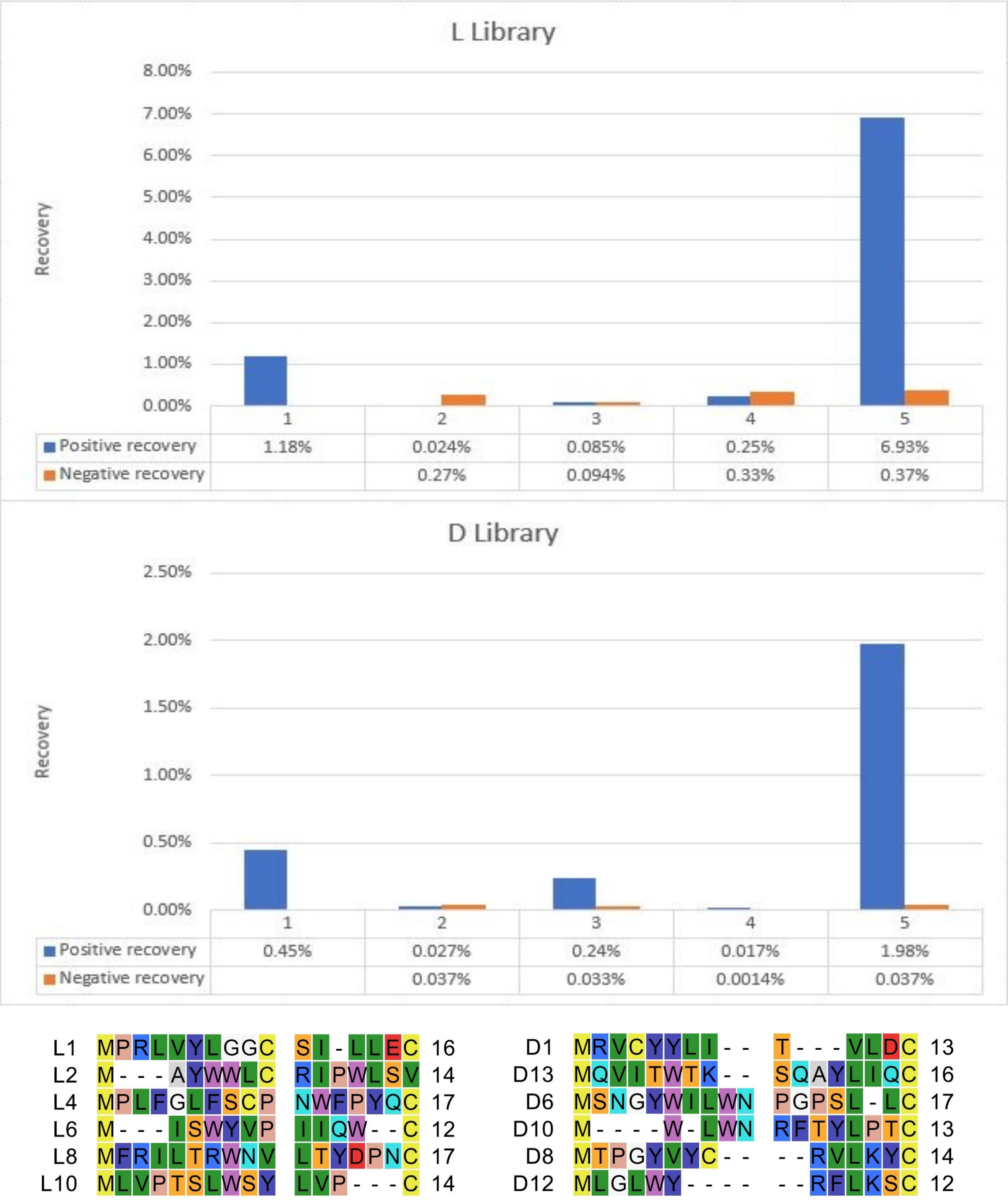

Figure S2. Selection results. *Upper*: Recoveries per round for both the L-Tyr and D-Tyr initiated libraries as determined by qPCR of output samples relative to input samples. Positive recoveries are shown with blue bars and represent recovery with immobilized GBA, while negative recoveries are shown with orange bars and represent recovery with the streptavidin beads alone. In both libraries, a spike in recovery can be observed after the 5th round, indicative of enrichment of binding peptides within the library and this was therefore selected to be further analysed. *Lower*: Alignment of sequences chosen from highest enriched unique sequences in the high-throughput sequencing of round 5 of each selection. Residues are coloured by type, and length is given after each sequence. Sequences are named by initiating amino acid stereochemistry and sequence abundance rank score (D1 being the highest abundance unique sequence from the library initiated with ClAc-D-Tyr).

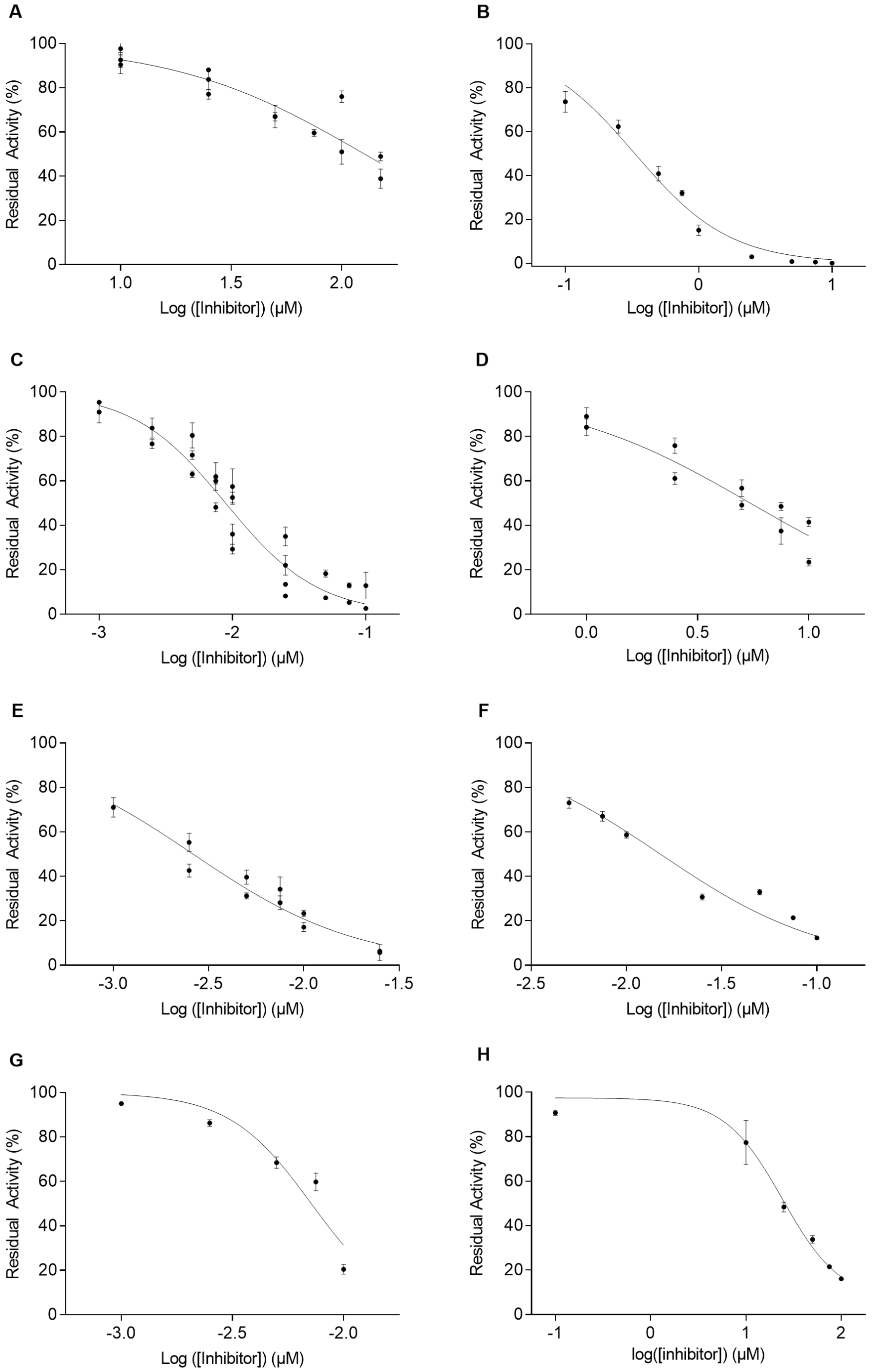

Figure S3. Inhibition curves using 4-MU-beta-glucoside as fluorogenic substrate. Residual rhGBA1 activity (%) *versus* the logarithm of different concentrations of inhibitors is plotted (See Table 1 for IC_{50} values). Inhibitors are (A) **L1**, (B) **L2**, (C) **L4**, (D) **L6**, (E) **D1**, (F) **D8**, (G) **D12**, and (H) **D13**.

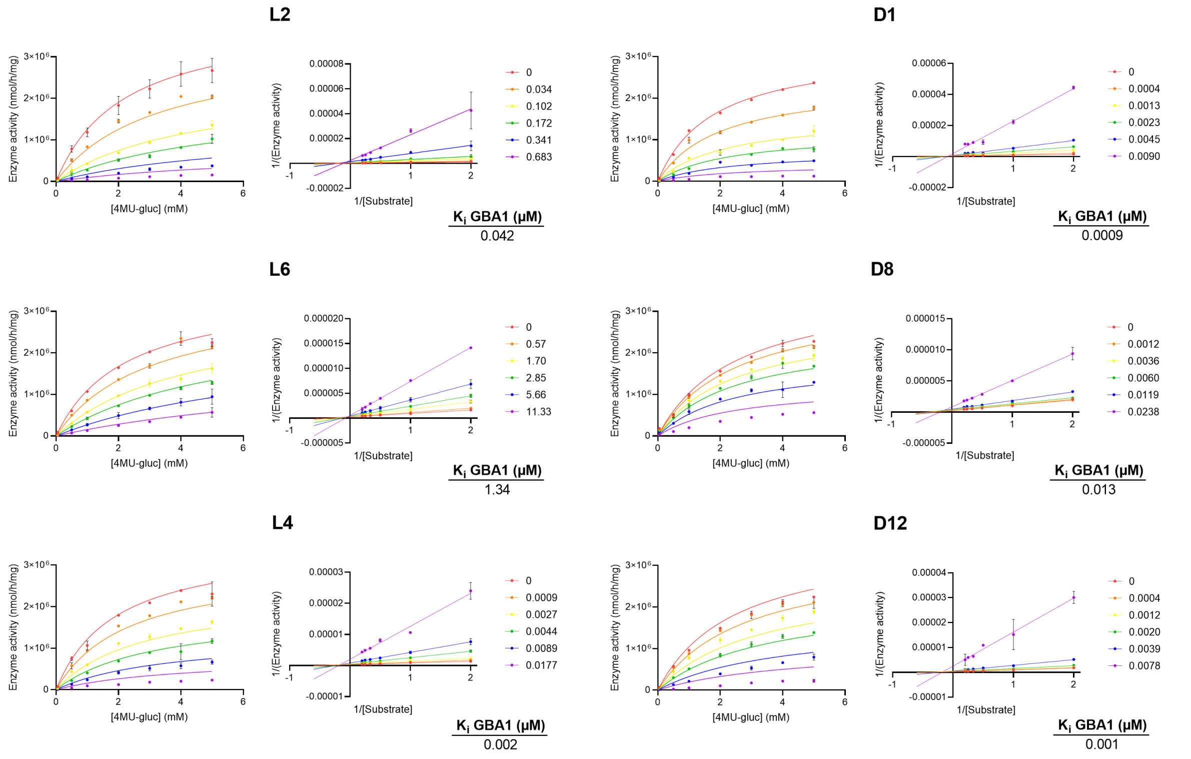

Figure S4. Inhibitory constant (K_i) values using increasing concentrations of 4-MU-beta-glucoside as fluorogenic substrate. Enzyme activity (nmol h^{-1} mg^{-1}) *versus* MU substrate concentrations is plotted (See Table 1 for K_i values) for inhibitors **L2**, **L4**, **L6**, **D1**, **D8**, and **D12**, and corresponding Lineweaver Burk plots are shown on the right.

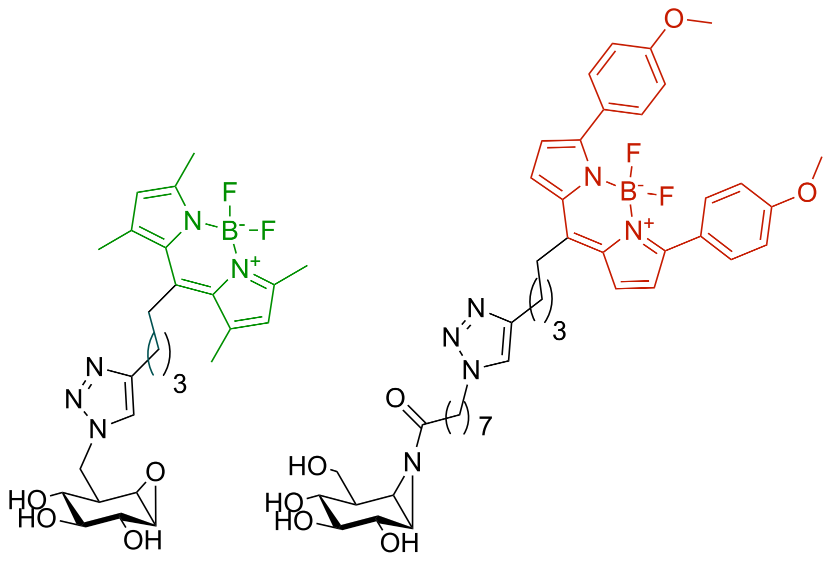

MDW393 **JJB75**

Figure S5. Chemical structures of activity-based probes (ABPs): **MDW393**[1] is a broad spectrum ABP targeting GBA1, GBA2 and GBA3, whereas **JJB75**[2] is selective for GBA1.

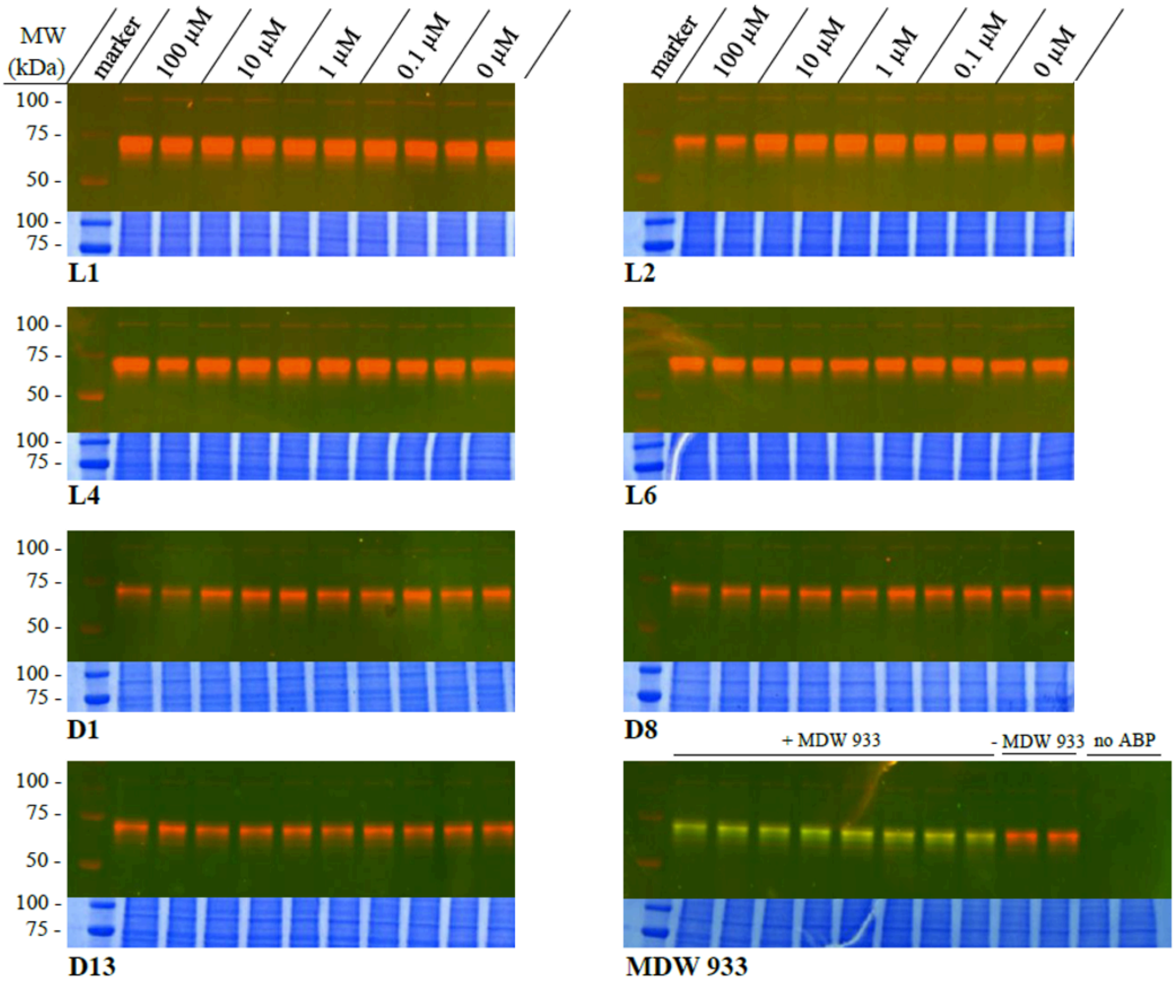

Figure S6. Competitive activity-based protein profiling (cABPP) to investigate cell permeability of the peptides. SDS-gels of RAW 264.7 cells treated with cyclic peptides (0, 0.1, 1, 10 and 100 µM) for 4 hours, and subsequently treated with broad spectrum Bodipy red GBA1, GBA2 and GBA3 ABP **JJB75** at 10 nM for 2 hours. Cells were washed, lysed and analysed by fluorescent readout. Bodipy green GBA1 ABP **MDW933** was used as positive control of a cell penetrant inhibitor.

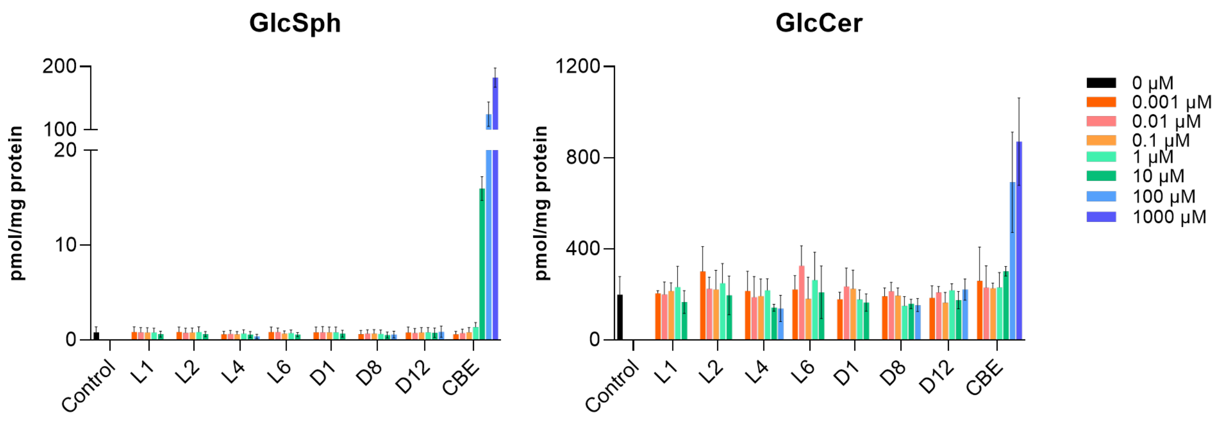

Figure S7. GlcSph and GlcCer levels produced by RAW 264.7 cells after treatment for 16 h with **L1**, **L2**, **L4**, **L6**, **D1**, **D8**, **D12** and **CBE** at consecutive concentrations. Values were plotted from n=2 replicates in technical duplos.

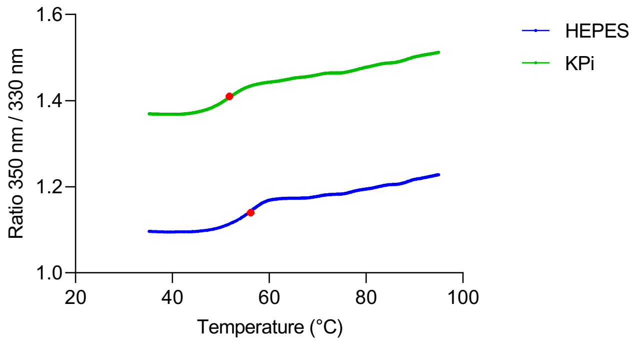

Figure S8. Melting curves of rhGBA1 (1 µM) in 10 mM KPi buffer pH 7,4 with 150 mM NaCl or 20 mM HEPES buffer pH 7,0 with 100 mM NaCl. Samples were incubated in the respective buffers for 30 minutes at room temperature.

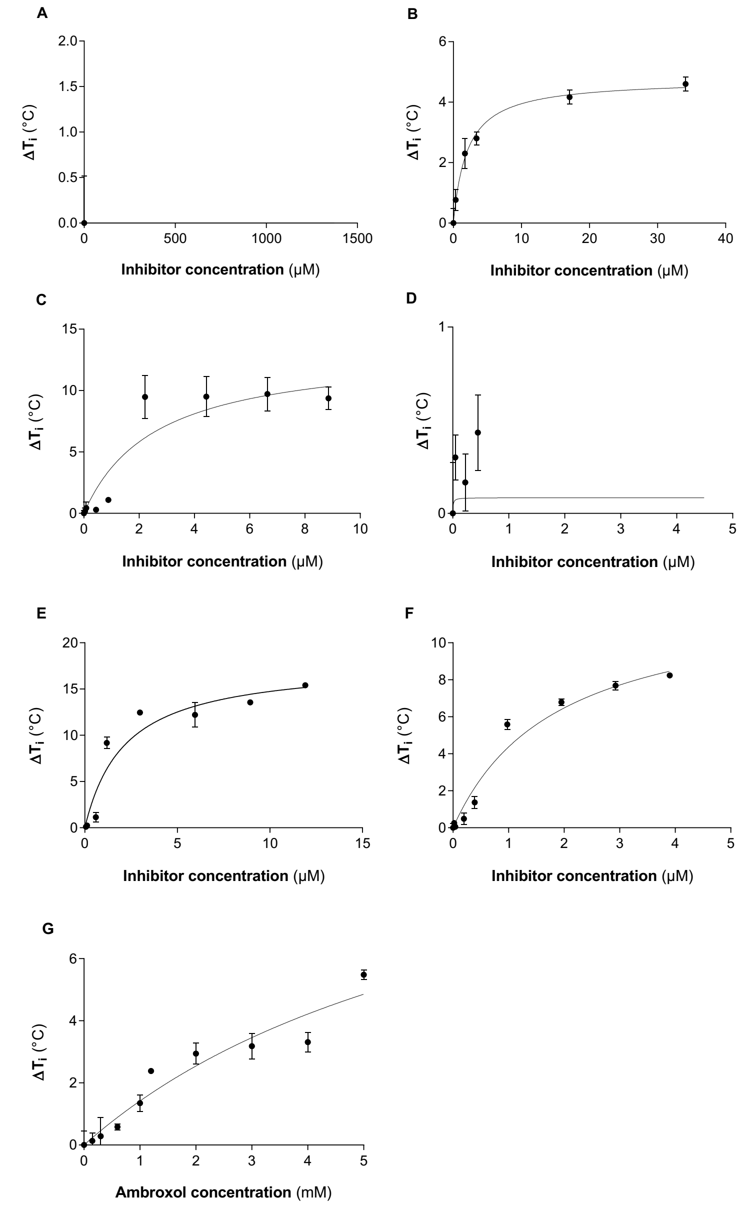

Figure S9. Melting curves of 1 µM rhGBA1 with increasing concentrations of peptides **L1** (A), **L2** (B), **L4** (C), **D1** (D), **D8** (E), **D12** (F) or Ambroxol (F). Samples were incubated at room temperature for 2 hours before measurement in the Tycho NT6.

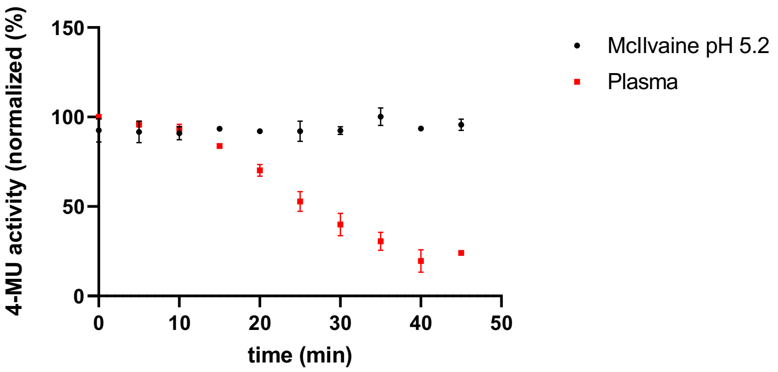

Figure S10. Cerezyme (10 nM) stability in McIlvaine buffer pH 5.2 or in plasma of a healthy individual. GBA1 activity measured in n=3 replicates, in technical duplicates.

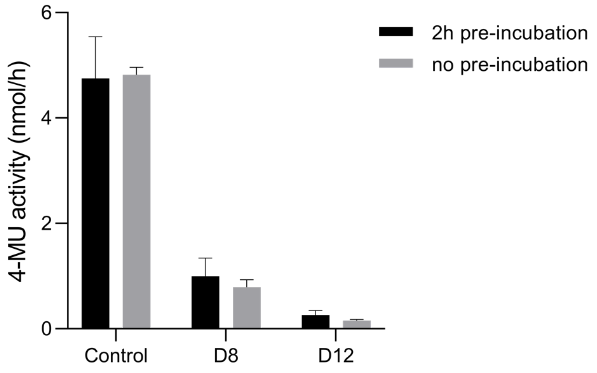

Figure S11. Intrinsic compounds stability of cyclic peptides **D8** and **D12** (0.1 µM) in plasma. rhGBA1 (10 nM) was added to compounds pre-incubated in plasma for 2h at 37°C or readily dissolved in DMSO, and incubated for 30 min, after which 4-MU activity was measured. Error ranges depict standard deviations from n=3 replicates, measured in technical duplicates.

Table S1. IC_{50} values for GBA1 inhibition in fibroblast lysates. Cells were treated with the peptides at increasing concentrations for 16 h, washed with PBS and homogenized. Reported values are mean ± standard deviation from 3 technical triplicates. N.D.: not determined.

Peptide	**GBA1 in fibroblast lysates**<br>IC_{50} (µM)
**L1**	39.7
**L2**	0.898
**L4**	0.067
**L6**	22.3
**D1**	0.440
**D8**	5.16
**D12**	0.334
**D13**	N.D.

2. Materials and Methods

2.1. Immobilisation of GBA on streptavidin beads

rhGBA1 (R&D systems) was biotinylated through addition of 40 equivalents biotin-PEG4-NHS for 60 hours on ice. Residual biotin probes were removed by buffer exchange with Zeba spin desalting columns (Thermo Scientific™) with the selection buffer (150 mM McIlvaine buffer + 0.1% triton-X100 + 0.2% sodium taurocholate). Immobilisation efficiency of the biotinylated rhGBA1 on Dynabeads™ M-280 Streptavidin (Invitrogen™) was estimated by immobilizing 2 pmol of biotinylated rhGBA1 with 0.5 - 8 µL bead slurry for 30 minutes at 4°C. Afterwards, the supernatants and immobilized fractions were collected and analysed with 12.5 % SDS-PAGE stained with SYPRO™ Ruby Protein Gel Stain (Invitrogen™) (Supplementary Figure S1).

2.2. Macrocyclic Peptide Library Design

DNA encoding the peptide library was assembled by PCR as previously described[3] to encode a T7 RNA polymerase transcription start site, a bacterial ribosome binding site, a start codon followed by 4–15 randomized residues encoded by NNK codons and terminated with a Cys-[Gly-Ser]$_3$ spacer and amber stop codon, and finally an annealing site for the puromycin-conjugated oligonucleotide. This DNA library was transcribed to RNA, ligated to puromycin, translated by *in vitro* translation, and reverse transcribed immediately before the selection against immobilized GBA.

2.3. RaPID selection

The RaPID macrocyclic peptide library was ligated to puromycin, *in vitro* translated at 2x100 µL scale (two separate libraries, initiated with ClAc-L-Tyr or ClAc-D-Tyr), and reverse transcribed as previously reported.[3] Afterwards, the library was buffer exchanged through a 1mL bed of G-25 sephadex (Cytiva) pre-swelled in selection buffer (150 mM McIlvaine buffer + 0.1% triton-X100 + 0.2 % sodium taurocholate). To this, the Saposin C 41-60 peptide was added to a final concentration of 100 µM. From here, a 0.5 µL sample was taken as an input measurement while the remainder was mixed with 200 nM final concentration of magnetic bead-immobilised GBA and was incubated at 4 °C while rotating for 30 minutes. These beads were thoroughly washed with selection buffer then cDNA of binding peptides was isolated by heating in 100 µL PCR buffer heated to 95 °C and transferring the supernatant to a new tube while hot. This was then quantified by qPCR referenced to a dilution series of reverse transcribed library (supplementary Figure S2) and amplified by PCR (cycle count set as qPCR Cq + 6). This amplified cDNA was subsequently transcribed into mRNA and the selection was continued using this new GBA binder-enriched library (using a 10 µL scale translation in round 2 and 5 µL from round 3 onwards). From the second round onwards, blocking buffer containing acetylated BSA and salmon sperm DNA was added to the peptide library following buffer exchange to limit nonspecific binding. In addition, three rounds of negative pulldowns with a 50/50 mixture of biotin-bound streptavidin and free streptavidin beads were done to eliminate peptides that bind to the magnetic beads. cDNA binding to the third set of negative beads was collected and quantified by qPCR to monitor the presence and enrichment of bead-binding peptides. Once enrichment of the library was observed after 5 rounds of RaPID selection (supplementary Figure S2), the cDNA was analysed by next-generation sequencing (Illumina MiSeq, following manufacturer's protocol) and selected peptides were identified.

To test individual hits, rather than a mRNA library only a single peptide was translated at the same scale as round 3+ selections. Furthermore, instead of 3 rounds of negative selections the desalted peptide solution was split in 2x 2.5 µL, where 1 was overlaid on positive selection beads and the other was overlaid on negative selection beads. Positive and negative selection beads were washed thrice with selection buffer and eluted as above. DNA was quantified by qPCR but not further amplified.

2.4. Peptide synthesis

Sapocin C 41-60 and lead peptide hits were synthesized using a Syro I automated peptide synthesizer (Biotage, Sweden) at 25 µmol scale, deprotecting with 40% piperidine for 3 minutes followed by 20% piperidine for 12 minutes at room temperature, then coupling with 4 equivalents each of amino acid, HOBt, and HBTU all added from stocks in DMF and 8 equivalents of DIPEA added from a stock in NMP for 40 minutes at room temperature. Resin was washed three times with DMF between each step.

After the final Fmoc deprotection step, resin was resuspended in 1 mL of a 0.2 M solution of ClAc-NHS in DMF and reacted with agitation at room temperature for 1 hour. Afterwards, all peptide beads were washed 5x with DMF and 5x with DCM and subsequently dried *in vacuo* for 30 minutes.

2 mL of a cleavage cocktail of trifluoroacetic acid (TFA) containing 2.5% each of ethane-1,2-dithiol (EDT), triisopropylsilane (TIPS), and water was added to the dried resin and the mixture was reacted with agitation for 3 hours at room temperature. The supernatant was then pushed through a filter into a 15mL falcon tube. The resin was subsequently washed 2 times with 1 mL of pure TFA, that was pushed through the filter into the same 15 mL tube. The cleaved peptides were then concentrated using a centrifugal evaporator for 60 minutes on low boiling point settings. After concentration the peptides were precipitated by addition of 10 mL ice-cold diethyl ether. The precipitated peptides were pelleted through hand-crank centrifugation for 30 seconds. The supernatant was discarded, and the peptides were washed 5x with 5 mL ice-cold Et_2O, mixing well, precipitating by hand crank centrifugation and discarding the supernatant each time. After washing the remaining Et_2O was evaporated through centrifugal evaporation for 5 minutes at low boiling point settings, followed by further drying *in vacuo* for 30 minutes. After drying the peptide pellet was resuspended in 4 mL DMSO and 15µL triethylamine (TEA) was added and the reaction mixture agitated for 1 hour at RT to cyclise. From this, 1 µL was diluted in 50 µL MeCN and analysed by MALDI-TOF to confirm cyclisation. Once cyclised, 15 µL TFA was added to neutralise the TEA. DMSO solutions of synthesized peptides were stored at -20°C prior to further purification by preparatory HPLC.

Final purification of the synthesized peptides was achieved by preparatory-HPLC using a C_{18} column with gradient elution from 10% MeCN +0.5% TFA up to 70% MeCN +0.5% TFA over 30 min. Fractions that exclusively contain the purified peptide were pooled and concentrated using centrifugal evaporation at HPLC settings for 3 hours. The concentrated peptide solution was then flash frozen in liquid N_2 and lyophilized overnight. Macrocyclic peptides were converted into HCl salts by dissolving the lyophilized peptides in 5 mL of 5 mM HCl + 0-3 mL MeCN, concentrating by centrifugal evaporation on HPLC settings for 3 hours, flash freezing in liquid N_2 and lyophilizing overnight, repeated 3 times. The purified, lyophilized peptides were dissolved in 20 µL DMSO and stored at -20°C.

Peptides L4, D8, and D12 were subsequently re-synthesised for further testing using a PurePep Chorus system (Gyros Protein Technologies, USA). Fmoc- amino acids were coupled for 15 min at 55°C on TG XV RAM resin (Rapp polymere) with 10 equiv. of DIC and 5 equiv. of Oxyma, capped with acetic

anhydride and pyridine for 5 min, deprotected for with 20% piperidine containing 0.1 M oxyma in DMF at 80°C for 1.5 min. Chloroacetic acid was included as any other building block, with omission of final Fmoc deprotection. Cleavage and global deprotection was by a 2 h reaction in a solution of 2.5 % EDT, 2.5 % TIPS, and 5 % water in trifluoroacetic acid (TFA), and the cleavage solution was subsequently added dropwise to a 20-fold excess amount of ice-cold diethyl ether to precipitate the product. The resulting precipitate was pelleted at 5000 rcf for 5 min, and the supernatant decanted. This pellet was washed by resuspending in 10 mL diethyl ether, centrifugation, and decanting of solvent as above for a total of three cycles. Residual diethyl ether was allowed to air dry. Cyclisation was performed by dissolving the dried crude peptide product to approximately 12.5 mM in DMSO and adding DIPEA until basic, then allowing the reaction to proceed at room temperature overnight before quenching with a slight excess amount of TFA. The macrocyclic peptides were subsequently purified by preparative-scale HPLC by using a Phenomenex (USA) Gemini C_{18} column (250 × 21.1 mm, 10 μm) with a 10–70 % gradient of acetonitrile in water (0.1 % TFA as additive for both) over 40 min at 12.5 mL min^{-1} with UV monitoring. Pure product fractions were pooled, lyophilized, and dissolved again in DMSO before concentration was determined by A_{280} using a sample diluted in phosphate buffer at pH 7.4 and extinction coefficients determined from the sequence using the Expasy ProtParam online tool (https://web.expasy.org/protparam/). A sample of the final purified product was analyzed by LC-MS on an Agilent 1260 II Infinity LC system using an Agilent poroshell-120 EC-C18 column (particle size: 2.7 μm, 100 x 4.6 mm) at a flow rate of 0.6 $mL.min^{-1}$ using a linear gradient of buffer A (0.1% Formic acid in H_2O) and buffer B (0.1% Formic acid in CH_3CN) from 5-95% B over 60 min, with detection by UV at 215 and 280 nm and Agilent InfinityLab LC/MSD XT in cycled positive and negative mode.

2.5. *In vitro* IC_{50} and K_i measurements

For GBA1, IC_{50} values were determined using the recombinant human GBA1 (rhGBA1, Cerezyme) following described procedures.[4] Enzyme activity was measured using 3.7 mM 4-methylumbeliferone (4MU)-β-D-glucopyranoside in 150 mM McIlvaine buffer pH 5.2 supplemented with 0.1% bovine serum albumin (BSA) (w/v), 0.1% Triton X-100 (v/v), and 0.2% sodium taurocholate (w/v). Varying concentrations of inhibitor (12.5 μL) were incubated with 12.5 μL of enzyme (final concentration 14 nM) at 37 °C for 30 min. Following this, 100 μL of 4MU substrate was added and the samples were again incubated at 37 °C for 30 min. The reaction was quenched using excess 1 M NaOH-glycine (200 μL, pH 10.3) and 4MU fluorescence was measured using a LS55 fluorimeter (λ_{Ex} 366 nm and λ_{Em} 445 nm).

GBA2 and GBA3 IC_{50} values were determined following a similar protocol,[5] using stable GBA2 or GBA3 HEK293T over-expression cell lysates and McIlvaine buffer pH 5.8 with 0.1 % BSA (w/v) and 100 mM HEPES with 0.1 % BSA, respectively. Covalent inhibitor of endogenous GBA1 (1 μM Bodipy green-cyclophellitol β-epoxide activity based probe) was added for both GBA2 and GBA3 assays, and GBA2 inhibitor *N*-(5-adamantane-1-yl-methoxy-pentyl)-deoxynojirimycin, AMP-DNM at 1 nM) was added for GBA3 assay. Cell lysates were pre-incubated with the aforementioned GBA1/GBA2 inhibitors for 30 min at 37 °C (12.5 μL) and subsequently incubated with the tested ligands at a range of concentrations for 30 min at 37 °C (12.5 μL), after which 100 μL 4MU-β-D-glucopyranoside was added for a final incubation of 30 min at 37 °C, quenched with 200 μL 1 M NaOH-glycine (pH 10.3) and fluorescence was measured. IC_{50} values were calculated using GraphPad Prism version 8.

GBA1 K_i values were determined using the same incubation times and buffers as described above following a previously described protocol.[6] 4MU-β-D-glucopyranoside concentration was varied between 0.05 mM and 5 mM. The inhibitors at different concentrations were not pre-incubated with the enzyme.

2.6. *In situ* cell studies

Human fibroblasts (Cambrex-Lonza CC-2511) were cultured in complete DMEM/F12 growth medium supplemented with 10% (v/v) foetal calf serum and 0.1 % (w/v) penicillin/streptomycin at 37 °C and 5% CO_2. Cells were seeded in 12-well plates during passage and the incubations were started when cells were 80% - 100% confluent. Peptides were added in 500 µL medium (10 and 50 µM final concentration) and incubated overnight. After incubation cells were washed three times with cold PBS and harvested in 30 µL lysis buffer (25 mM KPi, pH 6,5 supplemented with 0.1% Triton-X 100 (v/v) and protease inhibitor). Lysates were frozen at -80 °C until use. GlcCer and GlcSph lipids were quantified as described below. In parallel, enzyme activity in cell lysates was measured using 3.7 mM 4-methylumbeliferone (4MU)-β-D-glucopyranoside in 150 mM McIlvaine buffer pH 5.2 supplemented with 0.1% bovine serum albumin (BSA) (w/v), 0.1% Triton X-100 (v/v), and 0.2% sodium taurocholate (w/v) as described above (see Table S1).

RAW294.6 cells, cultured in RPMI with 10% (v/v) foetal calf serum, 1% (v/v) GlutaMAX, and 0.1 % (w/v) penicillin/streptomycin, were seeded at 0.8 × 10^6 cells per well in 12 well plates.

For competitive ABPP experiments, cells were incubated at confluency for 4 hours with inhibitors (0, 0.1, 1, 10 and 100 µM), and an additional 50 mM HEPES. Next, 10 nM broad spectrum Bodipy red GBA1, GBA2 and GBA3 ABP (**JJB75**) was added and incubated for 2 hours. Cells were washed thoroughly with PBS and lysed in ice cold KPi pH 6,5 with 0.1% Triton X-100 and protease inhibitor. Cells were kept on ice and equal protein amounts were loaded per well on a 10% SDS-PAGE gel. Gels were scanned for fluorescence and then stained with Coomassie Brilliant Blue for loading control.

For lipid levels determination, the cells were incubated at confluency with different concentrations of peptides and CBE as positive control for 16 h in 500 µL of medium. Cells were washed thoroughly with PBS and harvested in ice cold water. Lysates were kept at -80 °C until extraction.

2.7. 2.5. GlcCer and GlcSph lipid analysis

Levels of GlcCer and GlcSph were determined with a modified acidic Bligh & Dyer extraction (Figure 2 and S5).[7] Briefly, after addition of internal standards (C17-dh-Ceramide and $^{13}C_5$-Glucosylsphingosine), lipids were separated in an organic and an aqueous phase. The separated phases were dried under N_2 or in a concentrator. The dried organic lower phase was reconstituted in 0.1 M NaOH in MeOH and underwent microwave-assisted deacylation. After neutralization, the samples were dried. Both phases were further extracted and desalted with equal amounts of water and butanol. The butanol phases were taken up to dryness and the lipids were dissolved in MeOH and transferred to MS vials for analysis in a LC-MS/MS system as described previously.[7]

2.8. Thermostability assays in rhGBA (Cerezyme)

Varying concentrations of peptides were incubated with 1 µM rhGBA1 in 20 mM HEPES pH 7.0 with 100 mM NaCl, with a final DMSO concentration of 0.5 %, for 2 hours at room temperature. Melting

curves were determined in triplicate using the Tycho NT.6 (NanoTemper), according to manufacturer protocol.

2.9. Cerezyme Stabilization studies in plasma

Recombinant human GBA1 (rhGBA1, Cerezyme) was diluted to the final desired concentration of 10 or 25 nM in plasma from healthy individuals (Sanquin, The Netherlands) or 150 mM McIlvaine buffer pH 5.2 supplemented with 0.1% BSA (w/v), 0.1% Triton X-100 (v/v), and 0.2% sodium taurocholate (w/v). Time points were collected each 5 min in liquid nitrogen, up to 45 min for stability tests, and samples were kept at -80 °C until further processing.

The capacity of the peptides of interest to stabilize 10 nM of rhGBA1 in human plasma was tested with increasing concentrations (0.001 µM to 100 µM final concentration, with Ambroxol tested until 1000 µM). Samples were taken every 15 min, for a total time of 120 min. The snap frozen samples were kept at -80 °C until β-glucosidase activity analysis.

Specific GBA1 activity was determined as described above, with 25 µL of sample incubated for 30 min with 3.7 mM 4MU-β-Glc substrate.

3. Peptides Characterization

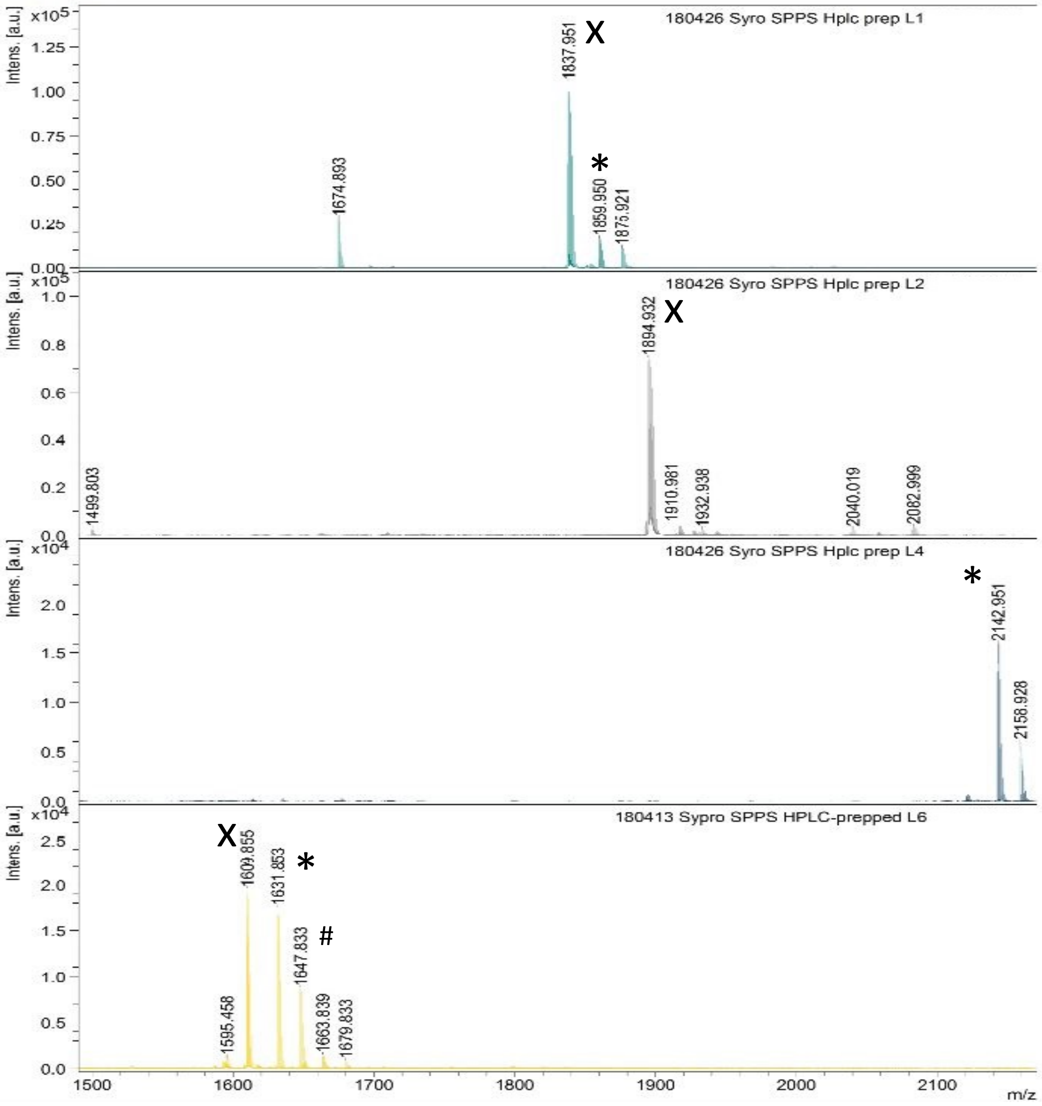

Figure S13. MALDI-TOF-MS spectra for purified peptides **L1**, **L2**, **L4**, and **L6**. Calculated monoisotopic masses are 1836.927, 1893.939, 2119.932, 1608.780 respectively. Identified product peaks are denoted with **X** for $[M+H]^+$, * for $[M+Na]^+$, and # for $[M+K]^+$.

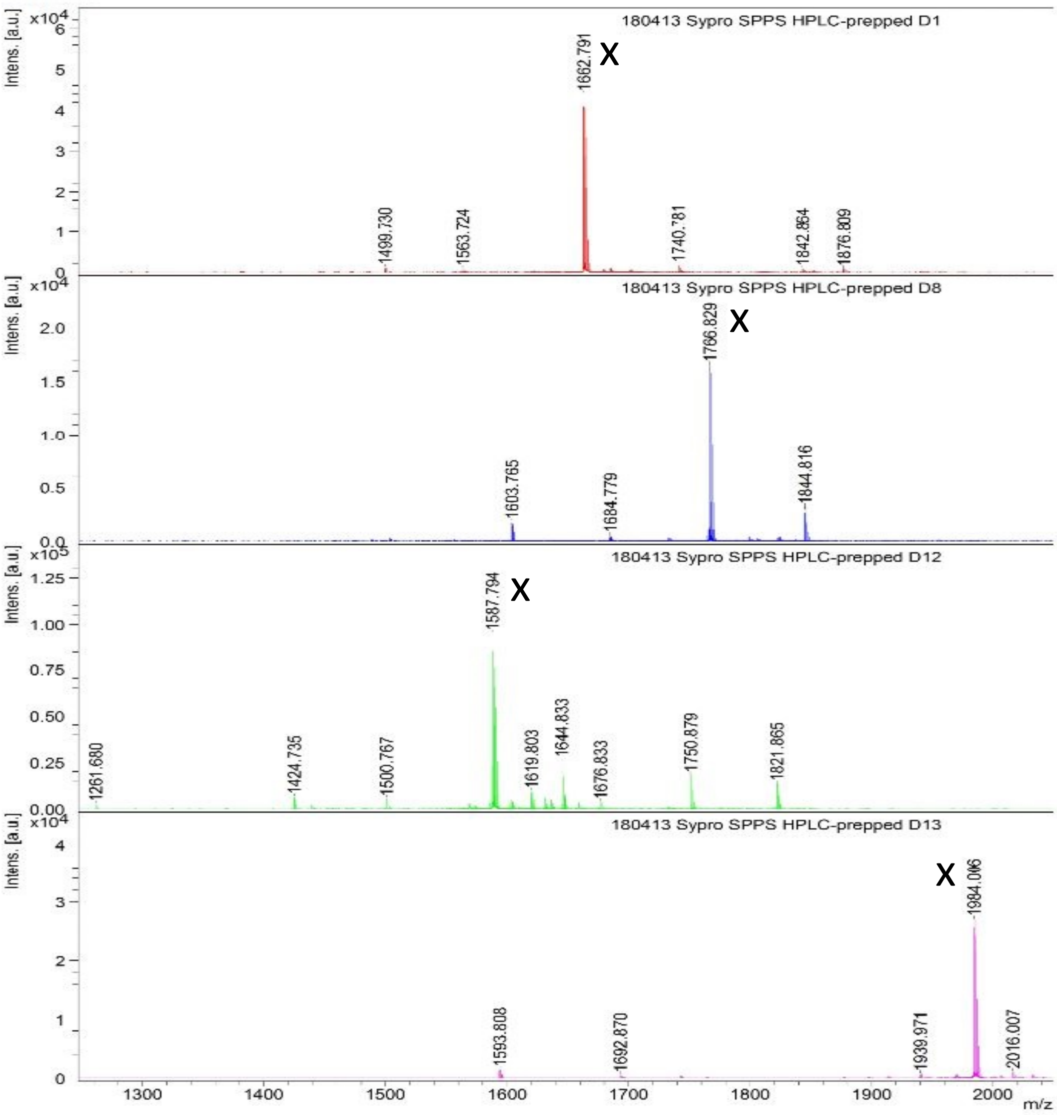

Figure S13 (continuation). MALDI-TOF-MS spectra for purified peptides **D1**, **D8**, **D12**, and **D13**. Calculated monoisotopic masses are 1661.795, 1765.832, 1586.807, 1982.992 respectively. Identified product peaks are denoted with **X** for $[M+H]^+$, * for $[M+Na]^+$, and # for $[M+K]^+$.

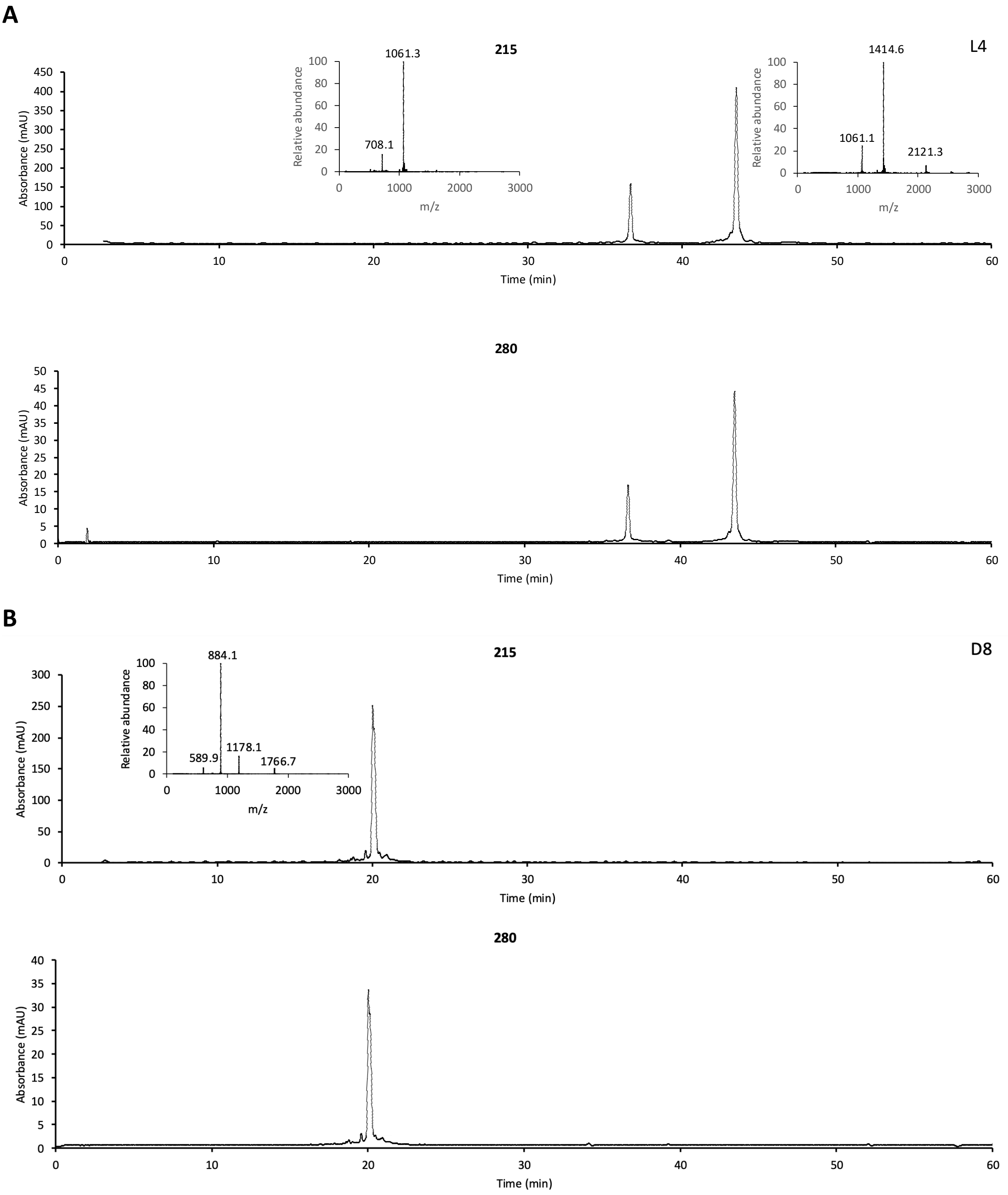

Figure S14. LC chromatograms at 215 and 280 nm and MS spectra of main peak (insets) for purified peptides following re-synthesis for stability testing. A) **L4**, calculated monoisotopic mass = 2119.932 Da, B) **D8**, calculated monoisotopic mass = 1765.832 Da.

C

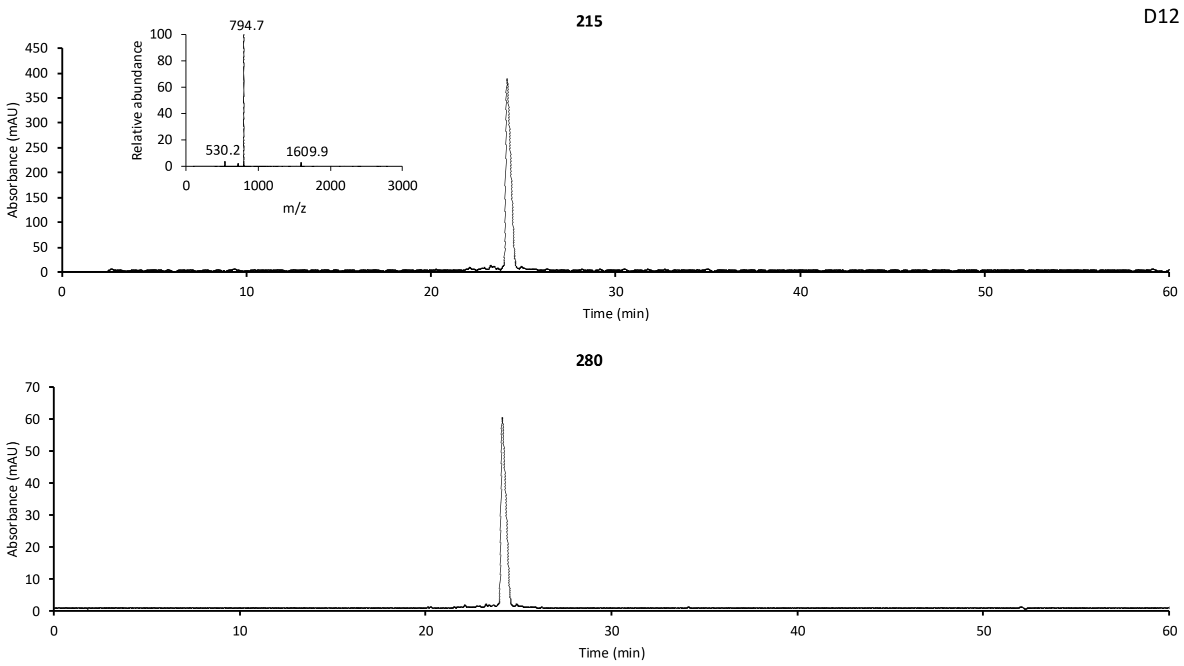

Figure S14 (continuation). LC chromatograms at 215 and 280 nm and MS spectra of main peak (insets) for purified peptides following re-synthesis for stability testing. C) D12, calculated monoisotopic mass = 1586.807 Da.

4. Raw gels images

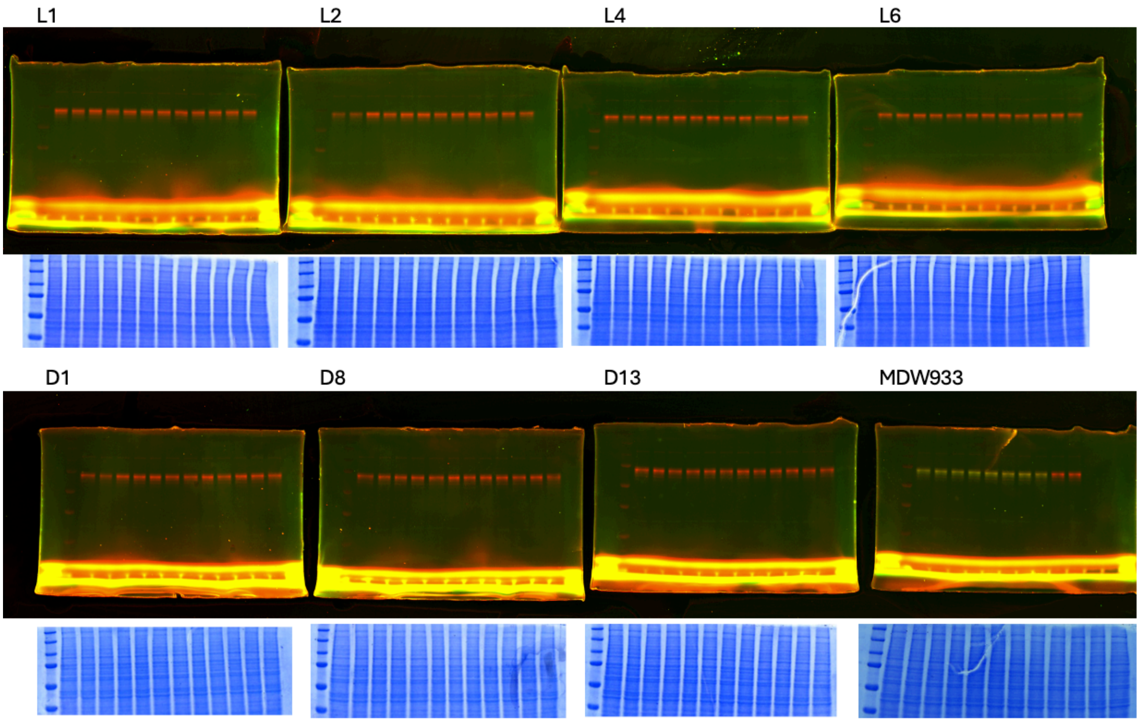

Figure S15. Scanned gels from Figure S4.

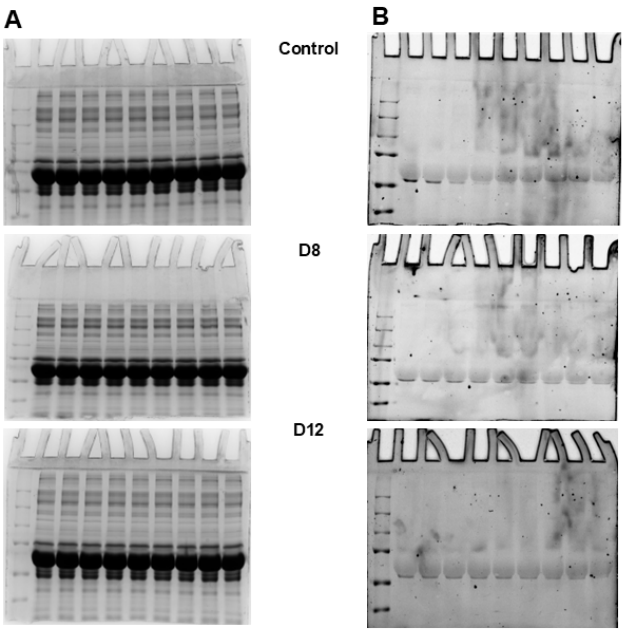

Figure S16. Scanned gels from Figure 3. A) Coomassie brilliant blue staining, as loading control and B) uncropped SDS-gels used Figure 4.

5. References

1 M. D. Witte, W. W. Kallemeijn, J. Aten, K. Y. Li, A. Strijland, W. E. Donker-Koopman, A. M. C. H. van den Nieuwendijk, B. Bleijlevens, G. Kramer, B. I. Florea, B. Hooibrink, C. E. M. Hollak, R. Ottenhoff, R. G. Boot, G. A. van der Marel, H. S. Overkleeft and J. M. F. G. Aerts, *Nat. Chem. Biol.*, 2010, **6**, 907–913.

2 K. Y. Li, J. Jiang, M. D. Witte, W. W. Kallemeijn, H. van den Elst, C. S. Wong, S. D. Chander, S. Hoogendoorn, T. J. M. Beenakker, J. D. C. Codée, J. M. F. G. Aerts, G. A. van der Marel and H. S. Overkleeft, *European J. Org. Chem.*, 2014, **2014**, 6030–6043.

3 J. Morimoto, Y. Hayashi and H. Suga, *Angew. Chem. Int. Ed. Engl.*, 2012, **51**, 3423–3427.

4 R. J. B. H. N. van den Berg, E. R. van Rijssel, M. J. Ferraz, J. Houben, A. Strijland, W. E. Donker-Koopman, T. Wennekes, K. M. Bonger, A. B. T. Ghisaidoobe, S. Hoogendoorn, G. A. van der Marel, J. D. C. Codée, H. S. Overkleeft and J. M. F. G. Aerts, *ChemMedChem*, 2015, **10**, 2042–2062.

5 Q. Su, S. P. Schröder, L. T. Lelieveld, M. J. Ferraz, M. Verhoek, R. G. Boot, H. S. Overkleeft, J. M. F. G. Aerts, M. Artola and C.-L. Kuo, *Chembiochem*, 2021, **22**, 3090–3098.

6 D. Lahav, B. Liu, R. J. B. H. N. van den Berg, A. M. C. H. van den Nieuwendijk, T. Wennekes, A. T. Ghisaidoobe, I. Breen, M. J. Ferraz, C. L. Kuo, L. Wu, P. P. Geurink, H. Ovaa, G. A. van der Marel, M. van der Stelt, R. G. Boot, G. J. Davies, J. M. F. G. Aerts and H. S. Overkleeft, *J. Am. Chem. Soc.*, 2017, **139**, 14192–14197.

7 M. Mirzaian, P. Wisse, M. J. Ferraz, A. R. A. Marques, P. Gaspar, S. V. Oussoren, K. Kytidou, J. D. C. Codée, G. van der Marel, H. S. Overkleeft and J. M. Aerts, *Clin. Chim. Acta*, 2017, **466**, 178–184.
